# A Tunable and Wearable Dual-Band Metamaterial Absorber Based on Polyethylene Terephthalate (PET) Substrate for Sensing Applications

**DOI:** 10.3390/polym14214503

**Published:** 2022-10-25

**Authors:** Qana A. Alsulami, S. Wageh, Ahmed A. Al-Ghamdi, Rana Muhammad Hasan Bilal, Muhammad Ahsan Saeed

**Affiliations:** 1 Chemistry Department, Faculty of Science, King Abdulaziz University, Jeddah 22254, Saudi Arabia; 2 Department of Physics, Faculty of Science, King Abdulaziz University, Jeddah 21589, Saudi Arabia; 3 Faculty of Electrical Engineering, Ghulam Ishaq Khan Institute of Engineering Sciences and Technology, Topi 23640, Pakistan; 4 School of Electrical Engineering, Korea University, Seoul 02841, Korea

**Keywords:** metamaterial, silver-nanoparticle, wearable, tunable, PET, absorber, Kapton, polymer

## Abstract

Advanced wireless communication technology claims miniaturized, reconfigurable, highly efficient, and flexible meta-devices for various applications, including conformal implementation, flexible antennas, wearable sensors, etc. Therefore, bearing these challenges in mind, a dual-band flexible metamaterial absorber (MMA) with frequency-reconfigurable characteristics is developed in this research. The geometry of the proposed MMA comprises a square patch surrounded by a square ring, which is mounted over a copper-backed flexible dielectric substrate. The top surface of the MMA is made of silver nanoparticle ink and a middle polyethylene terephthalate (PET) substrate backed by a copper groundsheet. The proposed MMA shows an absorption rate of above 99% at 24 and 35 GHz. In addition, the absorption features are also studied for different oblique incident angles, and it is found that the proposed MMA remains stable for θ = 10–50°. The frequency tunability characteristics are achieved by stimulating the capacitance of the varactor diode, which connects the inner patch with the outer ring. To justify the robustness and conformability of the presented MMA, the absorption features are also studied by bending the MMA over different radii of an arbitrary cylinder. Moreover, a multiple-reflection interference model is developed to justify the simulated and calculated absorption of the proposed MMA. It is found that the simulated and calculated results are in close agreement with each other. This kind of MMA could be useful for dual-band sensing and filtering operations.

## 1. Introduction

Metamaterials are artificially-engineered synthetic materials having periodic arrangements of sub-wavelength metallic/dielectric structures or resonators. In recent years, metamaterials have demanded a lot of recognition, due to their exotic and unique attributes such as negative-refraction, microwave cloaking, absorption of the inverse Doppler effect, etc. [1,2,3,4,5,6,7,8]. After the investigation of the first ‘perfect meta-absorber’ proposed in 2008 [4], MMAs have drawn significant interest from the microwave and optics community. These MMAs have multifarious features compared with the conventional Salisbury absorbers, such as a low-profile and light weight [9]. Moreover, they are thinner, more compact, and more efficient than conventional absorbers. They have many applications for wireless communication and other optical systems, including MIMO antenna isolation, EM interference reduction, stealth technology, and solar photovoltaics [10,11,12,13,14,15].

In the literature, several investigations have been carried out in this exciting area of MMAs from the microwave-visible spectrum [16,17,18,19,20,21,22]. Most of the reported work is based on rigid substrates without frequency spectrum reconfigurable abilities. The authors in [23] presented a multi-band metamaterial absorber fabricated on an FR4 substrate at the corresponding frequency points of 4.11, 7.91, 10.13, and 11.51 GHz. The metamaterial absorber reported in [24] contains wideband features having the operating range of 7–12.8 GHz, which is also developed on a rigid substrate. Similarly, different multi-band MMAs have been proposed in [16,25,26] which are also fabricated on rigid/solid substrates. Moreover, most of the present literature and investigations are based on the solid/rigid substrates [17,27,28] However, many applications required a flexible MMA with features such as the mitigation of multipath effects in a radome or the reducing of scattering noise in automotive radars. The authors in [29], going a step further, proposed a single-band tunable MMA ranging from 4.35 to 5.85 GHz. Such MMAs can have applications in software-defined radios. The authors in [30] presented single and dual-band absorbers at 77, 95, and 110 GHz on a flexible polyimide substrate. In [31], the authors proposed a dual-band flexible MMA with absorption peaks at 16.77 and 30.92 GHz.

Recent and modern communication systems and technological development in microwaves and optics require devices with multifarious features of miniaturization, cost-effectiveness, tuneability, and flexibility. To see contemporary advancement and evolution in the field of metasurfaces, a lot of research can be done to meet the modern standards of communication systems. Most of the recent meta-devices are fabricated on rigid and hard substrates, but the world is not flat and smooth: a lot of applications include curvilinear surfaces, conformal implementation, and wearable sensors [32], etc. where soft and flexible devices are needed [33]. Furthermore, modern communication systems demand efficient and multiband metasurfaces, to meet their future requirements. Therefore, the quest for flexible and tunable metasurfaces is inevitable.

Multi-band and tunable MMAs are well explored in the literature; however, a flexible MMA with the added functionalities of frequency reconfigurability at microwave frequencies has not been investigated yet. This paper bridges this gap by proposing a dual-band tunable MMA on a flexible PET substrate for communication applications such as MIMO antenna isolation, wearable sensors, and anti-jamming. The major contributions and novelty of this work are as follows: (1) a highly efficient dual-band MMA with an absorption rate of more than 99%. (2) Implementation of the dynamic tuning control on the proposed MMA by employing an active tuning element, i.e., a varactor diode. (3) The proposed MMA is designed on the flexible substrate of PET. (4) Finally, it is a bendable MMA with the additional features of frequency reconfigurability. Furthermore, the proposed research would be a valuable addition to the stream of tunable and conformable metasurfaces, and this also provides insight into implementing the smart meta-devices and reconfigurable intelligent surfaces (RISs) for future 6G communication applications.

The remainder of the paper is ordered as follows: Section 2 illustrates the literature survey on different kinds of metamaterial-based structures, including rigid, tunable, and flexible absorbers. The geometry and analytical part of the proposed metamaterial absorber are explained in Section 3. Section 4 discusses the different types of flexibility frameworks on which various kinds of microwave and optical devices can be wrapped to perform the bending analysis. The simulation results are briefly elaborated in Section 5. Multiple-reflection theory phenomena are highlighted in Section 6. Finally, the conclusion of the paper is elucidated in Section 7.

## 2. Literature Review

In this section, we briefly discuss the trends and state of the art of non-flexible, tunable, and flexible metamaterial absorbers.

### 2.1. Non-Flexible Absorbers

After the investigation of Landy’s seminal work on perfect metamaterial absorbers [5], there has been huge interest in the different microwave and optics communities in the field of metamaterial absorbers. In Landy’s initial work, they used a rigid/solid substrate of FR-4 to design the single and narrow-band absorbing device at 11.5 GHz. They also exploited and optimized the same unit cell to construct the metamaterial absorber for the terahertz band [34]. Thereafter, people started to explore metamaterial-based absorbers for dual-band, tri-band, multi-band, and wideband operation, targeting different operating spectrums including microwave, terahertz, visible and infrared [19,35,36,37]. Huang et al. investigated the tri-band perfect metamaterial absorber by designing three different sizes of square-shaped rings [38]. Afterward, Shen et al. also designed a tri-band perfect metamaterial absorber by using multiple resonators of different dimensions [39]. Furthermore, people started to study different techniques to implement multi-band and wideband metamaterial absorbers for different operating ranges. Usually, multi-layered- and multi-resonator-based configurations are used to enhance the bandwidth of the metamaterial-based absorber [27,39]. Shen and his co-workers arranged multiple layers in a vertical direction to enhance the absorption spectrum of metamaterial in the microwave regime [27]. A similar research group also designed the broadband metamaterial absorber in the terahertz band [40]. Moreover, people moved further and also explored the metamaterial-based absorbing structures in the visible and infrared spectrum. They used different types of plasmonic materials to explore the applications of metamaterial absorbers in solar photovoltaics and thermal emission. Hung et al. proposed an ultra-wideband metamaterial absorber for the visible light spectrum [41]. Cui et al. also presented the wideband metamaterial absorber for visible light applications [42]. All the aforementioned reported metamaterial-based absorbers have fixed operating frequencies and are designed/fabricated on rigid/solid substrates. Therefore, these features (fixed frequency and solid substrate) restrict their use to tunable and reconfigurable devices. Furthermore, these presented metamaterial absorbers cannot be used for cylindrical and spherical surfaces.

### 2.2. Tunable Absorbers

As we discussed earlier, due to the fixed operating frequency of most of the available metamaterial absorbers, they cannot be integrated/implemented with tunable and reconfigurable microwave and optical devices. Therefore, there is a strong need to study and investigate the design methods of tunable and reconfigurable metamaterials. A different type of tuning strategy can be used to implement the tunable metamaterials, depending on the operating spectrum of the discussed metamaterials [29,43,44,45,46]. In the microwave regime, active elements (PIN & varactor diodes) are used to tune the operating frequency and steer the beam of the metamaterial-based structure [29]. Zhao et al. investigated tunable metamaterial absorbers with the integration of a varactor diode. They tuned the absorption peak of the proposed metamaterial absorber by actively controlling the reverse bias voltage (capacitance) of the participating varactor diode [29]. In addition, a tunable metamaterial absorber is also presented in [43], which is designed for microwave frequencies. The operating frequency of this absorber is also reconfigured through the inclusion of a varactor diode in the geometry of the metamaterial. Furthermore, tunable metamaterial absorbers can also be developed for terahertz bands. In the terahertz band, usually, graphene and phase-change materials are employed to design tunable metamaterial-based devices [44,45,46]. Huang et al. proposed a tunable metamaterial absorber, by captivating the phase changeability of a dielectric material, strontium titanate (STO), which changes its permittivity with the influence of varying temperatures [44]. Next, Lei and his colleagues studied the thermally tunable metamaterial absorber by embedding a phase-change material, vanadium dioxide (VO2), which changes from an insulating to a metallic state with the variation of temperature [45]. A graphene-based tunable metamaterial absorber is also designed in [46] for the terahertz band. Moreover, for visible and infrared metamaterial absorbers, tunable features are introduced by incorporating the phase-change material, germanium-antimony-tellurium (GST), or liquid crystal [47,48].

### 2.3. Flexible Absorbers

In addition to the tunable functionalities of metamaterial-based absorbers, the latest and most contemporary communication systems, and the areas of industrial growth in microwave and optical devices involve devices with diverse features of miniaturization, cost-effectiveness, and flexibility. For designing and implementing the flexible metamaterial absorbers, plenty of flexible substrates such as polyamide, polymer, paper, rubber, Kapton, etc. are used [49]. Until now, several flexible meta-absorbers have also been investigated, ranging from GHz to THz frequencies. The authors implemented the inkjet-printed absorber based on the silver nanoparticle, mounted over a flexible substrate of paper. This flexible absorber was designed for the x-band of microwave frequencies, and manifests a narrowband absorption peak at 9.09 GHz [50]. Furthermore, Hao et al. [51] also studied the dual-band flexible metasurface absorber for THz frequencies. They used polyamide substrate as a flexible dielectric material to attain flexible features. In addition to these flexible absorbers, many applications including radars and stealth, etc., required stretchable/flexible metamaterial absorbers to absorb the unwanted EM signals [11,26]. Riad and his team [52] designed the THz flexible absorber for infrared stealth applications. Similarly, Krzysztof et al. also implemented the flexible metamaterial absorber for the THz band. In this work, a simple and easily fabricable ring resonator was imprinted on the flexible substrate of polyimide [11]. Similarly, Tao et al. designed the narrow-band THz absorber, which worked on a single operating point of 1.6 THz [53]. By adopting similar methods, a highly efficient flexible metamaterial absorber can be designed for any desired operating frequency.

## 3. Geometry, Analytical Treatment and Simulation Setup

### 3.1. Theory of Metamaterial Absorbers

The general principle of the absorber is based on the transmission theory of EM waves. The input impedance of the MMA should be perfectly matched with the impedance of the incoming EM wave incident on the MMA. Due to the impedance matching, the EM waves are not reflected, and they are trapped in the lossy dielectric, resulting in enhanced absorption. The imaginary part of the refractive index of the MMA is made as large as possible, due to which the EM waves are absorbed in the dielectric material. The absorption of the MMA can be calculated using the following equations [21,54]:

(1) $$A\left(\omega \right)\;=\;1\;-\;R\left(\omega \right)-T\left(\omega \right)$$

(2) $$A\left(\omega \right)\;=\;1\;-\;{\left|S_{11}\left(\omega \right)\right|}^{2}\;-\;{\left|S_{21}\left(\omega \right)\right|}^{2}$$

where *A*(ω) is the absorption, $R\left(\omega \right)$ is the reflection and $T\left(\omega \right)$ is the transmission of the MMA. Since the ground plane is fully coated with the metal in the device configuration of MMA, the transmission is zero. The absorption is calculated using the reflection coefficient expressed in terms of the scattering parameter.

(3) $$A\left(\omega \right)\;=\;1\;-\;{\left|S_{11}\left(\omega \right)\right|}^{2}$$

### 3.2. Design of Meta-Unit Cell

**Figure 1:**
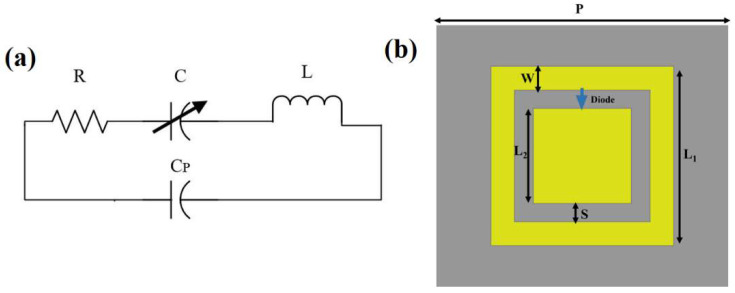
Design specification of the proposed MMA: (**a**) RLC equivalent circuit model of the varactor diode, and (**b**) schematic of the meta-unit cell of the proposed MMA.

**Figure 2:**
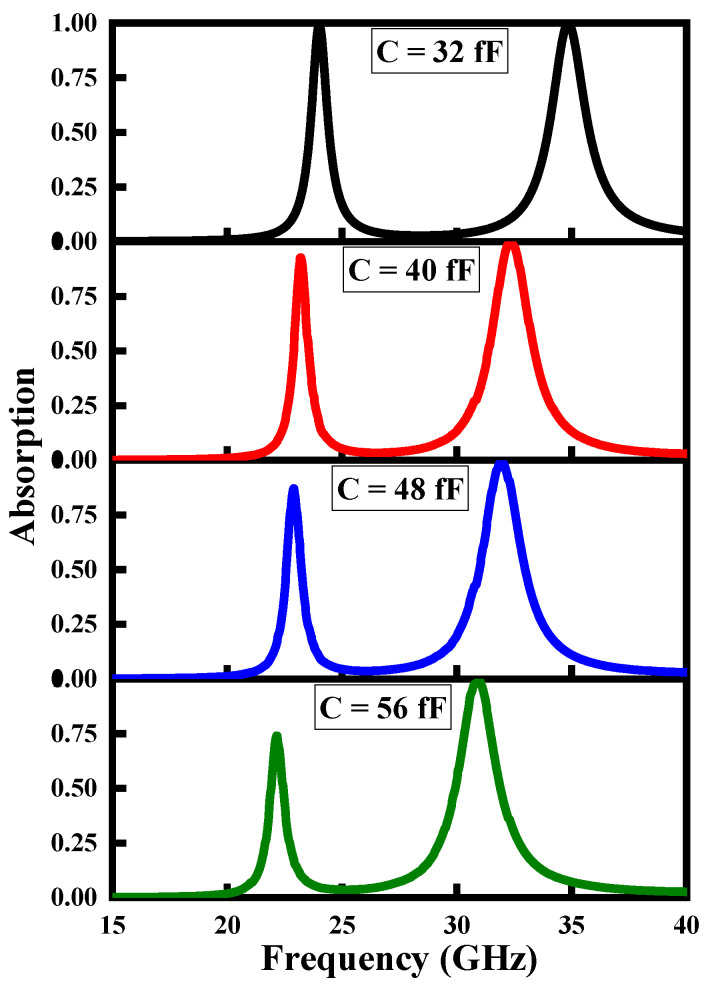
Tuning mechanism of absorption rate of the proposed MMA at different capacitance values of the varactor diode; here, C shows the value of the capacitance of the varactor diode.

The unit cell [38] of the proposed tunable and flexible MMA is shown in Figure 2, and is considered due to its simplicity and is inspired by the previous work [55]. The unit cell consists of a metallic patch with a metal square ring at the outside mounted over a 0.27 mm thick PET substrate. The two absorption peaks correspond to the two meta-rings, present in the main unit cell. The lower-frequency peak is owing to the outer square ring, and the higher-frequency peak originates due to the inner square patch. The dielectric constant ($\varepsilon_{R}$) and the loss tangent (tanδ) of the PET substrate are 2.9 and 0.019, respectively. The bottom ground layer is made up of copper with a conductivity (σ) of 5.8 × 10^7^ S/m. Silver is used for the top layer. The optimized design parameters of the proposed MMA are as follows: P = 3 mm, L_1_ = 2.31 mm, L_2_ = 1.5 mm, W = 0.205 mm, S = 0.16 mm. For tuning and reconfigurability features, active elements, a PIN diode, and a varactor diode can be used. Active elements are available in many configurations, but their integration in the metamaterial-based structures depends on the operating frequency spectrum. For the present work, the tunability of the unit cell is achieved by placing a MACOM varactor diode model MAVR-011020-1411 [56] between the inner patch and outer ring, which has a dynamic range of capacitance from 32–216 fF. PIN diodes can also be used for tuning purposes, but have only two states (ON or OFF). Conversely, the varactor diode has multiple states, which give us more freedom to tune the spectral characteristics of the proposed MMA. The resonance frequency of the unit cell changes by changing the capacitance of the varactor diode. The equivalent circuit model of the used varactor diode is illustrated in Figure 1a, where R, L and C represent the resistance, inductance, and variable capacitance of the varactor diode, respectively. Here, R is fixed, considered as 4 Ω, and L is taken as 0.4 nH. The proposed MMA is simulated using CST Microwave Studio software. The optimized geometric parameters of the proposed MMA are shown in Figure 1b.

## 4. Flexibility Framework

Usually, conventional electronic and optical devices are implemented on rigid and non-flexible substrates, which make them unsuitable for advanced integrated optoelectronic devices. Modern devices including antennas and metasurfaces are fabricated on flexible substrates, to make their integration and implementation possible where we need to mount flexible devices on cylindrical and non-flat surfaces. Therefore, in this section, we discuss all the possible flexibility frameworks or bending operating conditions at which the desired metasurface or flexible device can be wrapped or rolled over an arbitrary uniform or non-uniform shape, i.e., a cylinder, sphere, cone, etc. The flexibility framework can be categorized in various ways, such as

Structural bending

Non-structural bending

Uniform bending

Non-uniform bending

Control bending

Uncontrolled bending

Here, we briefly discuss the details of the most commonly-used bending environment (structural, uniform, and non-uniform), one by one.

### 4.1. Cylindrical Bending

In this type of working environment, a flexible metamaterial is bent or folded over an arbitrary cylinder of different radii to predict its spectral characteristics. This type of environment lies in the domain of structural and uniform bending. To precisely observe the performance stability of the required flexible device, we bend the examined flexible device for different values of an arbitrary cylindrical shape. From a practical point of view, there are many applications where we mount the flexible device on the cylindrical surface like a human arm, pillars, or walls in buildings. For example, for on-body applications, a flexible antenna is rolled on a human arm, to observe its efficiency and stability. In large buildings, to efficiently and intelligently reflect the signals or steer the beams in the desired location, we implemented the flexible reflective intelligent metasurfaces on the different pillars of the buildings.

### 4.2. Spherical Bending

This section deals with the spherical bending of flexible devices or metamaterials. In this type of bending, a flexible device is bent on an arbitrary sphere, for its bending analysis to be performed. The spherical bending analysis is very useful for various applications, including radar absorbers based on flexible metamaterials and reflect-array antennae for breast cancer applications. This kind of bending condition is also classified as structural and uniform bending.

### 4.3. Conical Bending

Conical bending can also be performed for a more rigorous analysis of flexible devices or metamaterials. This type of bending analysis can be considered as structural and non-uniform bending. Under these operating conditions, a flexible metamaterial is wrapped over a cone of a non-uniform radius, to observe its performance under a harsh environment.

## 5. Simulation Results and Discussion

In this section, the spectral characteristics of the proposed MMA are analyzed and studied under different operating environments. The operating frequency of the traditional MMAs is fixed, and cannot be changed after fabrication. On the other hand, tunable MMAs can be used for a wide range of frequencies, which makes them more desirable for practical application. Figure 2 illustrates the absorption rates of the proposed MMA at different capacitance values of the varactor diode. Absorption features of the designed MMA are inspected by varying the capacitance of the varactor diode ranging from 32–56 fF; the reason for choosing this specific set of capacitance values is the better performance of the MMA in this dynamic range. As we vary the capacitance of the proposed MMA from 32 fF to 56 fF, the two absorption peaks manifest red-shift in their operating range, i.e., $f\;=\;1/2\lambda \sqrt{LC}$, where L and C are the inductance and capacitance values of the varactor diode, respectively. The lower operating band shows a gradual decrease in its absorption peak, while the higher operating band maintains its absorption value.

**Figure 3:**
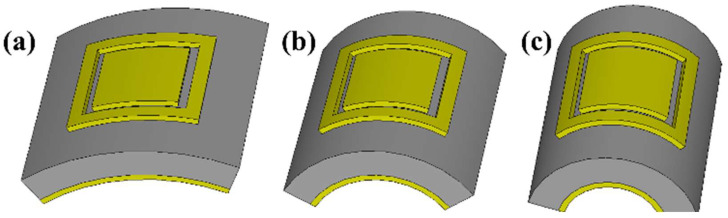
Schematic diagram of bending analysis of the proposed MMA over the radii of different arbitrary cylinders, (**a**) R = 10 mm, (**b**) R = 2 mm, and (**c**) R = 1.5 mm. In this fig., the smaller the value of the radius (R), the larger the bending condition.

**Figure 4:**
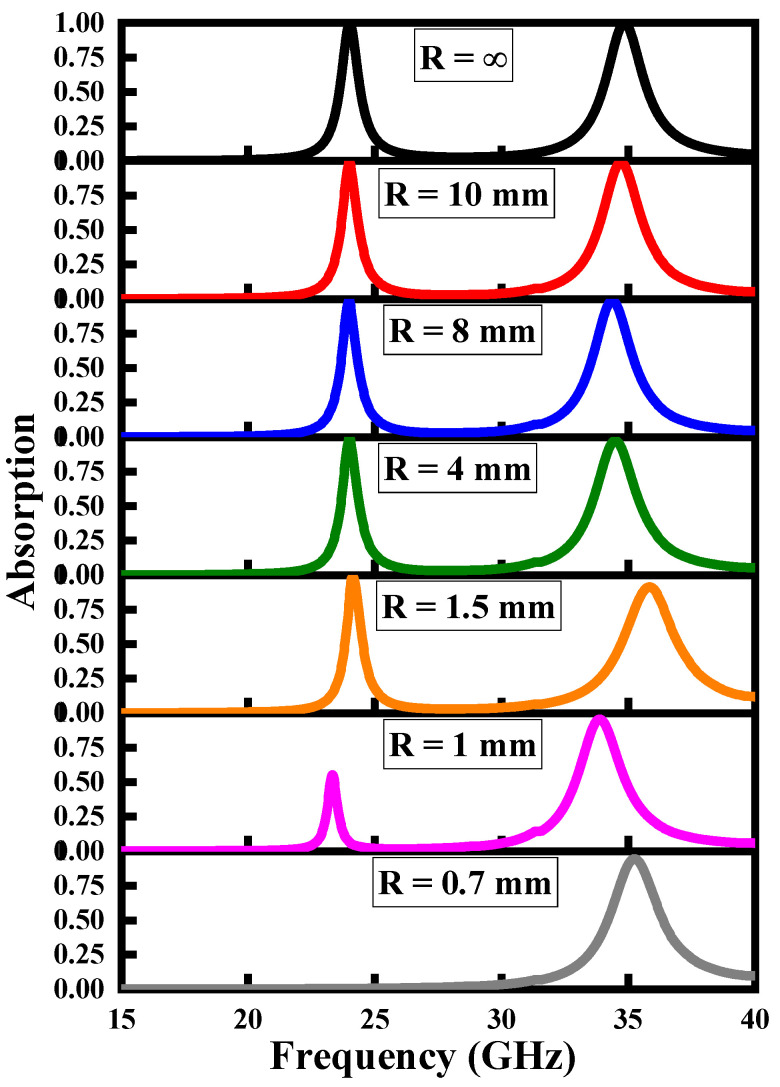
Bending analysis of the absorption rate of the proposed MMA at different radii of an arbitrary cylinder. R = ∞ shows no bending conditions, whereas R = 0.7 mm indicates extreme bending conditions.

The flexibility and conformability of the proposed MMA give it many advantages over the traditional MMAs, such as lighter weight, more durability, and easy integration onto unusual surfaces such as spheres or cylinders. The MMA bending over different radii is shown in Figure 3, and the respective absorption performance spectra are given in Figure 4. The extreme bending limit can be estimated from P = 2πR, where P is the periodicity of the device and R is the radius of an arbitrary cylinder. The absorption of the proposed MMA remains unchanged (except for the operating frequency of the higher operating band) when it is wrapped over an arbitrary cylinder of different radii, namely R = ∞, 10 mm, 8 mm, 4 mm, and 1.5 mm. When the MMA is bent over the small radii of an arbitrary cylinder, the absorption value of the lower operating band starts decreasing because the outer square ring vanishes (is compressed) within an extreme bending condition. Based on this bending analysis of the proposed MMA, it can easily be integrated onto cylindrical and spherical surfaces, and find applications in flexible devices and wearable sensors.

**Figure 5:**
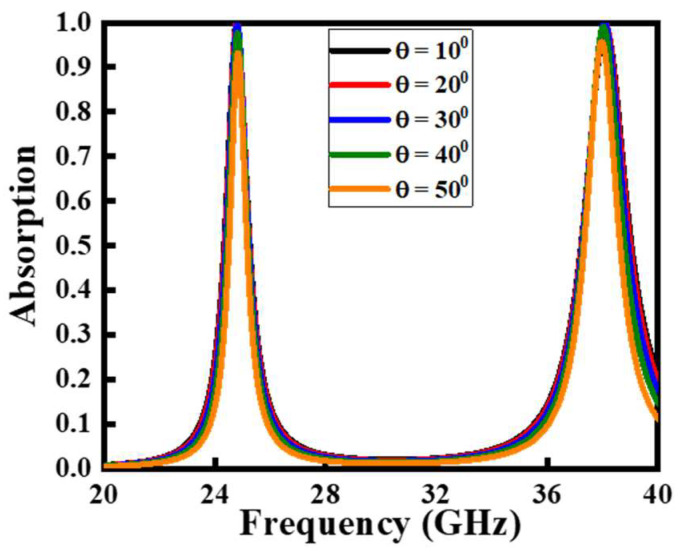
The absorption rate of the proposed MMA at different incidence angles of the EM waves. θ represents the incident angle of the incoming EM waves.

**Figure 6:**
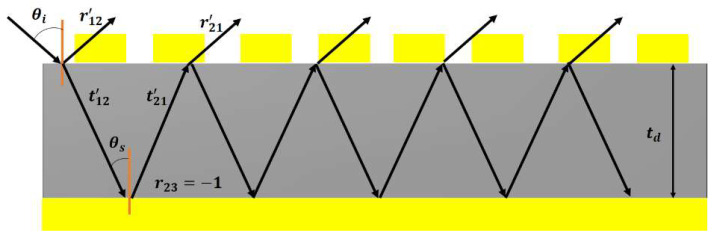
Illustration and schematic of the multiple-reflection model of the proposed MMA.

Angle insensitivity is very important for MMA, since the EM waves can strike the MMA surface at any incident angle. Therefore, it is very necessary to analyze the angular robustness of the proposed MMA under the influence of different incident angles. Figure 5 illustrates the absorption rates of the designed MMA at different incident angle θ, where θ is the angle between the *z*-axis and the propagation vector. It can be seen from Figure 6 that the absorption rate is greater than 90% under the inspection of various incident angles from 10–50°. Therefore, it can be predicted that the proposed MMA remains robust for the oblique incident angles of the incident EM waves.

Table 1 highlights the absorption characteristics of proposed MMA over the bending of different radii of an arbitrary cylinder, under the stimulus of different states of a varactor diode. For better analysis, and under the impact of bending for different varactor states, we will discuss a few cases step by step.

Initially, we consider the case R = 1 mm, C = 56 fF. The absorption value of the lower operating band is only 11%. When we tune the C = 48 fF by keeping the bending fixed (R = 1 mm), the absorption starts to increase to 22%. Similarly for C = 40 fF and C = 32 fF, keeping the bending fixed means the absorption increases to 37% and 55% respectively. However, the absorption is increases, but the operating frequency of the lower band shows a blue shift in its operating spectrum. Considering the situation R = 2 mm, C = 56 fF, the MMA demonstrates an absorption value of 64% at the lower operating band. When we change the C = 48 fF by keeping the bending fixed (R = 2 mm), the absorption starts increasing to 78%. Similarly, for C = 40 fF and C = 32 fF, keeping the bending fixed, the absorption increases to 90% and 99% respectively. Based on this analysis, the decrease in the absorption peaks can be adjusted by carefully stimulating the state of a varactor diode.

## 6. Interference Theory and Analytical Calculation

In this section, a multiple-reflection theory-based scheme is presented to analyze the transmitted and reflected waves inside the dielectric spacer which is supposed to function as a cavity model. The top periodic antenna arrays perform the function of impedance-tuning surfaces, and the lower ground sheet is assumed as a zero-thickness metallic plate, and behaves as a reflector. When the incoming EM waves strike the top periodic arrays, it penetrates the spacer, due to the impedance matching phenomena. Thereafter, multiple-reflection phenomena are induced inside the dielectric spacer, as shown in Figure 6. Their corresponding mathematical quantities can be written as: $\overset{´}{r_{12}}\;=\;r_{12}e^{i\varphi_{r12}}$ and $\overset{´}{t_{12}}\;=\;t_{12}e^{i\varphi_{r12}}$. The transmitted component collides with the lower ground reflector and reflects the dielectric spacer with the reflection amplitude of −1. It has a complex propagation phase $\;\beta \;=\;nk_{o}t_{d}$, where $t_{d}$ and $k_{o}$ are the thickness of the substrate and free-space wavenumber, respectively. Again, partial transmission-reflection occurs with the relevant energies of $\overset{´}{t_{21}}\;=\;t_{21}e^{i\varphi_{r21}}$ and $\overset{´}{r_{21}}\;=\;r_{21}e^{i\varphi_{r21}}$, respectively. Owing to these multi-reflection steps, destructive interference happens. Finally, the total reflection can be expressed in the following expression, as in [57]:

(4) $$r\;=\;\overset{´}{r_{12}}\;-\;\frac{\overset{´}{t_{12}}\overset{´}{t_{21}}e^{i2\beta }}{1\;+\;\overset{´}{r_{21}}e^{i2\beta }}.$$

**Figure 7:**
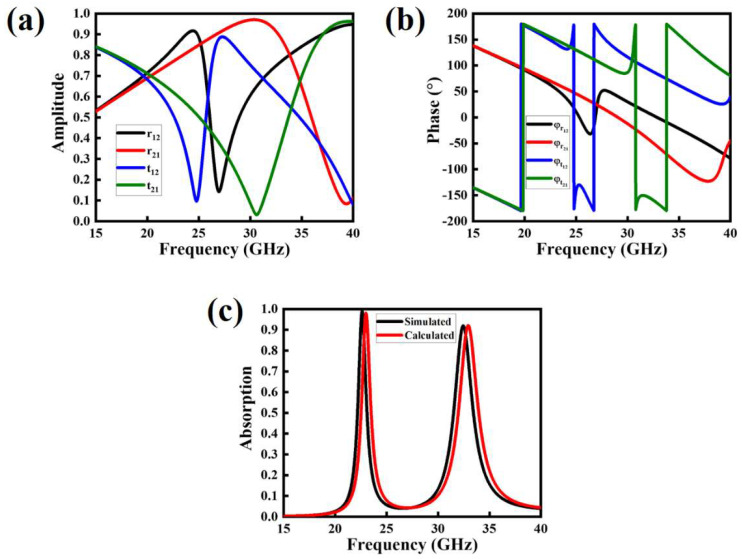
Estimated results of the proposed MMA through multiple-reflection-based interference theory model, (**a**) amplitude of the reflected and transmitted waves inside the cavity model, (**b**) phase of the reflected and transmitted waves inside the cavity model, and (**c**) comparative analysis of simulated and calculated absorption.

Thus, the overall absorption of the proposed MMA can be approximated by $A\;=\;1\;-\;{\left|r\right|}^{2}$. Figure 7a,b depict the magnitudes and phases of the transmitted and reflected light at the air-spacer interface, which is calculated by employing the interference theory model. Figure 7c compares the simulated and calculated absorption curves of the proposed MMA, and it is noted that both plots are in good agreement with each other.

## 7. Conclusions

In this work, we presented a dual-band flexible MMA with the added functionality of tunable features. The MMA demonstrates an absorption rate of more than 99% at the corresponding microwave frequencies of 24 and 35 GHz. Furthermore, the proposed MMA manifests an angle-insensitive absorption response under the influence of the different incident angles of the incoming EM waves. The frequency tunability characteristics are attained by integrating a varactor diode between the two resonating elements. We observed a red-shift in operating frequency by varying the capacitance of the varactor diode, ranging from 32–56 fF. Finally, the absorption properties were also investigated by bending the designed MMA over an arbitrary cylinder of different radii, and it was found that the absorption remained unchanged for R = 1.5–∞. In addition, the proposed absorber can also be extended to tri and quad bands by adding one or two extra outer rings, respectively. This kind of MMA could be useful for various applications, namely MIMO antenna isolation, wearable sensors, and anti-jamming.

## Figures and Tables

**Table 1:** Detailed bending analysis of the proposed MMA for different states of the varactor diode in tabulated form: there are four small tables, namely top left, top right, bottom left, and bottom right, etc. All the tables indicate the bending of the proposed MMA by fixing the varactor state for each case. In addition, Table 1 consists of different symbols, which are explained at the bottom of the table.

Varactor State, C = 32 fF					Varactor State, C = 40 fF				
B	ALP	AHP	LOP	HOP	B	ALP	AHP	LOP	HOP
R = ∞	100	100	24.04	34.93	R = ∞	99	92	22.916	32.629
R = 10	100	100	24.025	34.85	R = 10	93	98	23.30	32.60
R = 8	100	100	24	34.5	R = 8	93	98	23.30	32.55
R = 4	99	97	24.025	34.62	R = 4	93	97	23.30	32.525
R = 2	99	94	24.07	34.62	R = 2	90	94	23.325	32.45
R = 1.5	98	91	24.20	36	R = 1.5	85	93	23.325	32.50
R = 1	55	95	23.25	34.025	R = 1	37	97	22.65	32.375
R = 0.7	0	93	X	35.40	R = 0.7	0	98	X	33.325
**Varactor State, C = 48 fF**					**Varactor State, C = 56 fF**				
R = ∞	99	91	22.396	31.576	R = ∞	86	87	21.358	29.704
R = 10	86	98	22.70	31.925	R = 10	74	98	22.10	31.125
R = 8	88	98	22.80	32.125	R = 8	76	97	22.15	31.225
R = 4	87	96	22.725	31.925	R = 4	76	96	22.225	31.30
R = 2	78	94	22.625	31.55	R = 2	64	93	22.025	30.90
R = 1.5	78	93	22.975	32.475	R = 1.5	62	93	22.425	31.75
R = 1	22	98	22.10	31.475	R = 1	11	98	21.45	30.525
R = 0.7	0	99	X	32.275	R = 0.7	0	100	X	31.175

R = radius of a cylinder at which the absorber has been wrapped, C = capacitance of a varactor diode, X = operating point of lower band where absorption is zero, B = bending, ALP = absorption at a lower point, AHP = absorption at a higher point, LOP = lower operating point, HOP = higher operating point.

## Data Availability

All the relevant data are presented in this research article, but may be obtained from the authors upon reasonable request.
